# The Evolutionary Divergence of *psbA* Gene in *Synechococcus* and Their Myoviruses in the East China Sea

**DOI:** 10.1371/journal.pone.0086644

**Published:** 2014-01-23

**Authors:** Qiang Zheng, Nianzhi Jiao, Rui Zhang, Jingjing Wei, Fei Zhang

**Affiliations:** State Key Laboratory for Marine Environmental Science, Institute of Marine Microbes and Ecospheres, Xiamen University, Xiamen, People’s Republic of China; Beijing Institute of Genomics, Chinese Academy of Sciences, China

## Abstract

Marine *Synechococcus* is a principal component of the picophytoplankton and makes an important contribution to primary productivity in the ocean. Synechophages, infecting *Synechococcus*, are believed to have significant influences on the distribution and abundance of their hosts. Extensive previous ecological studies on cyanobacteria and viruses have been carried out in the East China Sea (ECS). Here we investigate the diversity and divergence of *Synechococcus* and their myoviruses (Synechomyoviruses) based on their shared photosynthesis *psbA* gene. *Synechococcus* is dominated by subclades 5.1A I, 5.1A II and 5.1A IV in the ECS, and clades I and II are the dominant groups in the Synechomyoviruses. As two phylogenetically independent clades, there is much higher diversity of the Synechomyoviruses than *Synechococcus*. Obvious partitioning characteristics of GC and GC3 (the GC content at the third codon position) contents are obtained among different picophytoplankton populations and their phages. The GC3 content causes the *psbA* gene in *Synechococcus* to have a higher GC content, while the opposite is true in the Synechomyoviruses. Analyzing more than one-time difference of the codon usage frequency of *psbA* sequences, the third position nucleotides of preferred codons for *Synechococcus* are all G and C, while most Synechomyoviral sequences (72.7%) have A and T at the third position of their preferred codons. This work shed light on the ecology and evolution of phage-host interactions in the environment.

## Introduction

Marine picocyanobacteria, mainly including *Synechococcus* and *Prochlorococcus*, and photosynthetic picoeukaryotes are the principal primary producers in the ocean [1]–[3]. Cyanophages, the viruses infecting cyanobacteria, control the mortality of their hosts and participate in nutrient regeneration and cycling in the ocean [4]–[7].

Marine *Synechococcus*, an ancient and genetically diverse clade, is ubiquitous in the global ocean and is abundant in both estuarine and coastal waters [8]–[10]. The genus has developed many eco-types to adapt to different environments, and is divided into three major subclusters, 5.1, 5.2 and 5.3 [9], [11]. Synechomyoviruses (*Synechococcus* myoviruses) infect and interact with their hosts in the marine environment, leading to the rapid diversification for both of them [12]. Synechomyoviruses are known to encode accessory metabolic genes, including those for photosynthesis (*psbAD*) and carbon metabolism (*talC, zwf, gnd, cp12*), which are obtained via horizontal gene transfer (HGT) and constitute a large reservoir of the genetic diversity pool [6], [13]–[23].

Recently, photosynthetic genes (*psbA*) coding for key photosystem II proteins (D1) have been widely discovered in the genome of cyanomyoviruses and also frequently detected from marine metagenomic data [13], [15], [18], [24]–[26]. The *psbA* gene provides a shared gene marker between hosts and phages to investigate their antagonistic co-evolution in the marine environment. The GC contents in *psbA* sequences from Synechomyoviruses (46%–51%) are lower than those from their *Synechococcus* hosts (56%–62%) [27]. Synechomyovirus *psbA* sequences have a patchy GC distribution as a result of intragenic recombination [27], [28]. Since the *psbA* gene has been obtained by cyanophages for a long time and evolved into an independent clade, the question arises as to how cyanophages diverged the genes which were obtained via HGT.

The East China Sea (ECS) is one of the largest continental marginal sea in the world, and is heavily influenced by human activity and multi-currents, such as the Yangtze dilution water, the Kuroshio current, the Taiwan warm current and so on [29]–[32]. Studies on distribution patterns and abundance about cyanobacteria and viruses have been extensively carried out over the past decades in the ECS [29], [30], [33]–[36]. However, little is known concerning the diversity and community structure of the cyanobacteria and cyanophages in this complex oceanic area. The purpose of this study was to illuminate the diversity and evolutionary divergence of the *psbA* gene in *Synechococcus* and their myoviruses in the ECS.

## Results and Discussion

### Community Structure

**Figure 1:**
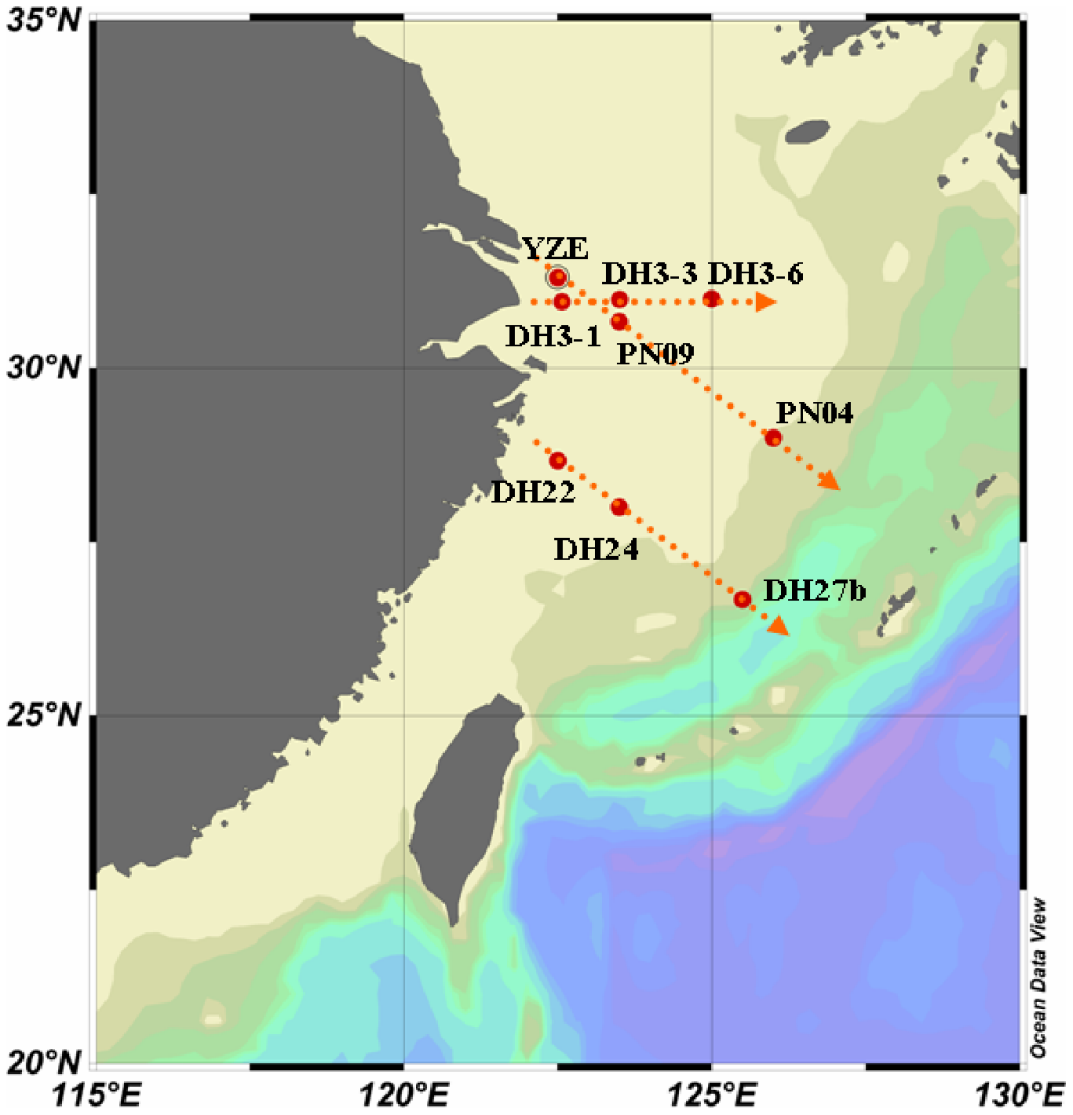
Map of the ECS showing the locations of the sampling stations.

In summer, the estuarine station DH3-1 had more than one half of its clone sequences belonging to photosynthetic eukaryotic picophytoplankton (PEP), but the number was greatly decreased with increasing distance off-estuary (**Fig. 1**). Station DH3-1 also had the most Synechomyoviruses and the least *Synechococcus* sequences in this transect (**Table 1**). This might have been caused by viral aggregation or attachment to particles, which would then block the filters. In stations DH3-3 and DH3-6, *Synechococcus* sequences dominated. No sequence of *Prochlorococcus* or their phages was found in these three stations.

**Table 1:** The community composition in each station.

Taxon	DH3-1	DH3-3	DH3-6	DH22	DH24	DH27b-5M	DH27b-150M	PN09	PN04	Total	GOS
S	7	81	93	0	0	5	0	9	2	197	283(96)
SM	36	13	2	40	29	13	0	73	71	277	282(123)
SP	3	1	0	0	0	0	0	1	2	7	-
PEP	48	1	0	48	62	11	2	15	9	197	-
P	0	0	0	3	5	69	98	2	13	190	-
Total	94	96	95	91	96	98	100	100	97	868	

S, *Synechococcus*; SM, *Synechococcus* Myovirus; SP, *Synechococcus* Podovirus; PEP, photosynthetic eukaryotic picophytoplankton; P, *Prochlorococcus* and *Prochlorococcus* phages.

In the nearshore-offshore transect (DH22-DH24-DH27b), *Synechococcus* sequences were only found in station DH27b-5M (**Fig. 1**). However, the Synechomyovirus sequences declined with offshore direction. PEPs also showed the same trend and were the predominant community in the two near-shore stations (**Table 1**). *Prochlorococcus* and their phages were detected in all stations, but increased significantly in the open-shelf station DH27b (both DH27b-5M and DH27b-150M). In the euphotic zone (150 m) of station DH27b, all sequences were *Prochlorococcus* and their phages except for two PEP clones (**Table 1**).

In the cross-shelf PN transect, no PCR product was obtained from the nearest estuarine station YZE (**Fig. 1**). This indicated that all the communities containing the *psbA* gene had a low abundance in winter near the estuary. From PN09 to PN04, *Synechococcus* and PEPs sequences decreased, and Synechomyovirus sequences basically remained the same (**Table 1**). Although many more sequences of *Prochlorococcus* and their phages were acquired from station PN04 compared to PN09, the proportion was much lower than at station DH27b.

### The Diversity of *Synechococcus psbA* Sequences

**Figure 2:**
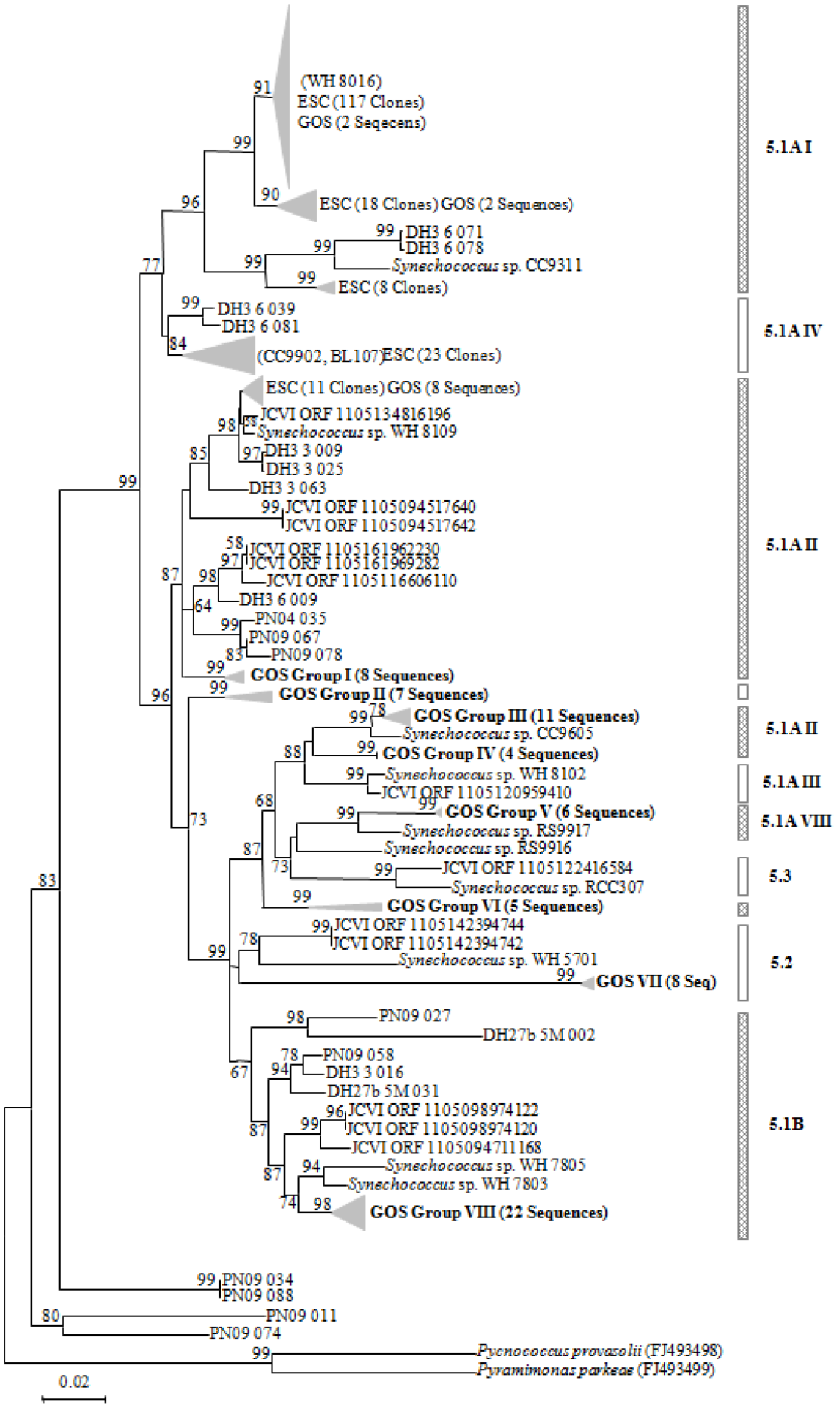
The neighbor-joining tree based on the *Synechococcus psbA* sequences. Bootstrap percentages (>50) are shown in the tree. Scale bar represents 2% nucleotide substitution percentage.

In the ECS, *Synechococcus* was dominated by subclusters 5.1A, in which clades I, II and IV were the predominant components. Notably, the clade 5.1A I took up nearly three quarters of all *Synechococcus* sequences (**Fig. 2**). Sequences from the GOS database formed eight small clades, which might be related to their specific original habitats. As a result of the uneven sequences obtained from the different stations, we ignored their diversity analyses along the transect.

### The Diversity of *psbA* Genes in Synechococcus Myoviruses

**Figure 3:**
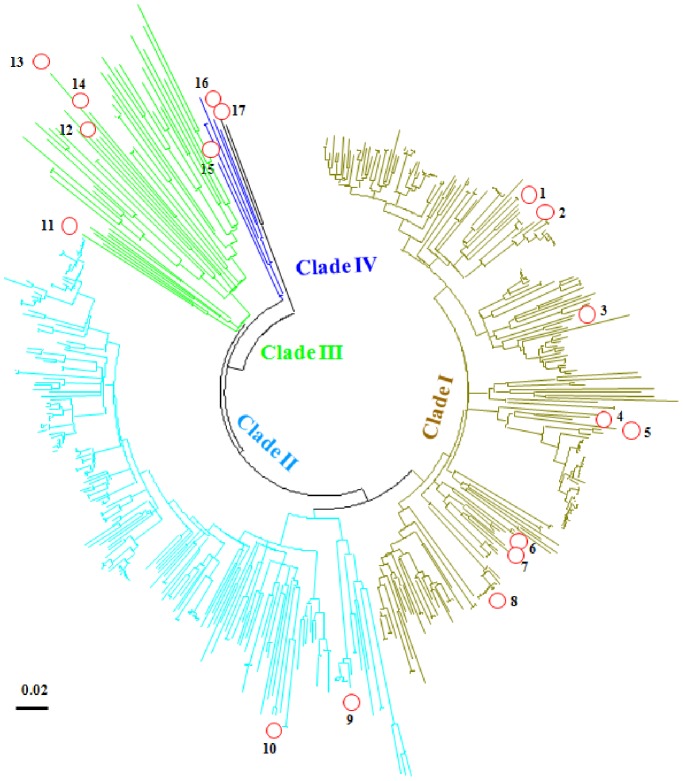
The neighbor-joining phylogenetic tree for all Synechomyovirus *psbA* sequences. 1. S-SM1 (GU071094); 2. S-RIM8 (JF974288); 3. S-SM2 (GU071095); 4. Syn33 (GU071108); 5. Syn1 (GU071105); 6. Syn19 (GU071106); 7. S-RSM4 (FM207411); 8. Syn9 (DQ149023); 9. S-ShM2 (GU071096); 10. S-SSM5 (GU071097); 11. S-PM2 (AJ630128); 12. S-CRM01 (HQ615693); 13. S-ShM1 (); 14. S-SSM7 (GU071098); 15. Cyanophage S-TIM5 (JQ245707); 16. *Pycnococcus provasolii* (FJ493498); and 17. *Pyramimonas parkeae* (FJ493499). Scale bar represents 2% nucleotide substitution percentage.

All Synechomyovirus could be classified into four groups - clades I, II, III and IV, in which clades I and II are the dominant groups in the ECS (**Fig. 3**). These clades are inconsistent with their host classification. However, Cyanophage S-TIM5 represents a previously unknown lineage of myoviruses [37], and here its *psbA* sequence also formed a unique clade (IV) (**Fig. 3**).

**Figure 4:**
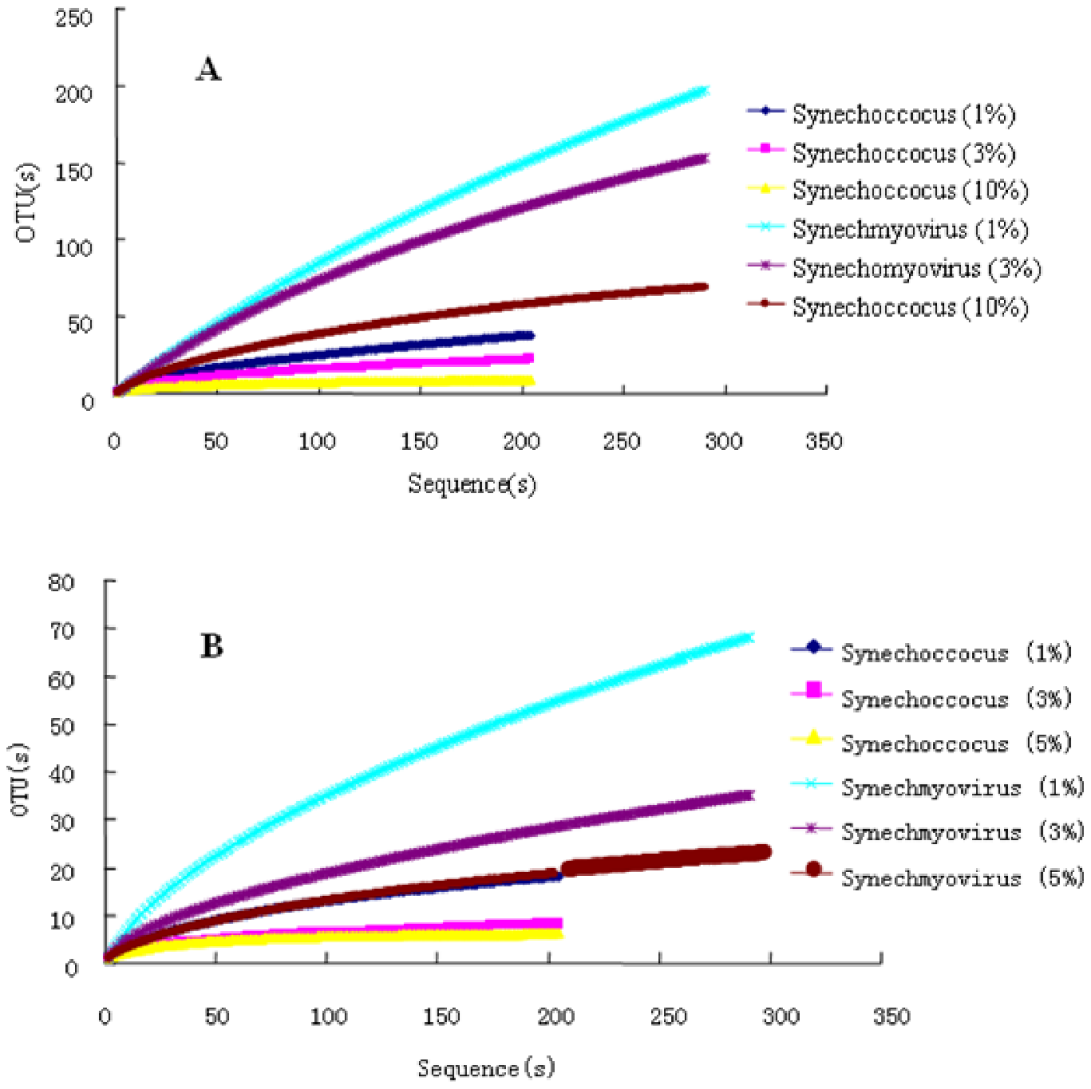
Rarefaction curves. Based on A: nucleotide sequences; B: amino acid sequences. Cutoff values are selected as 1%, 3% and 10% for nucleotide sequences, and 1%, 3% and 5% for amino acid sequences.

The diversity of *psbA* sequences belonging to *Synechococcus* phages was far higher than that of their hosts, even though the sampling method did not target the phages (**Fig. 4**). This indicated that viral *psbA* genes are not only undergoing an independent selection but also evolving under an increased evolutionary rate [27], [28].

### Partitioning Based on GC and GC3 Content

Studies show that the GC content can separate *psbA* sequences into *Prochlorococcus* and their phages (39%–46%), *Synechococcus* phages (46%–51%) and *Synechococcus* (56%–62%) [27]. We added GC3 content (GC content at the third codon positions) to analyze our *psbA* sequences.

**Figure 5:**
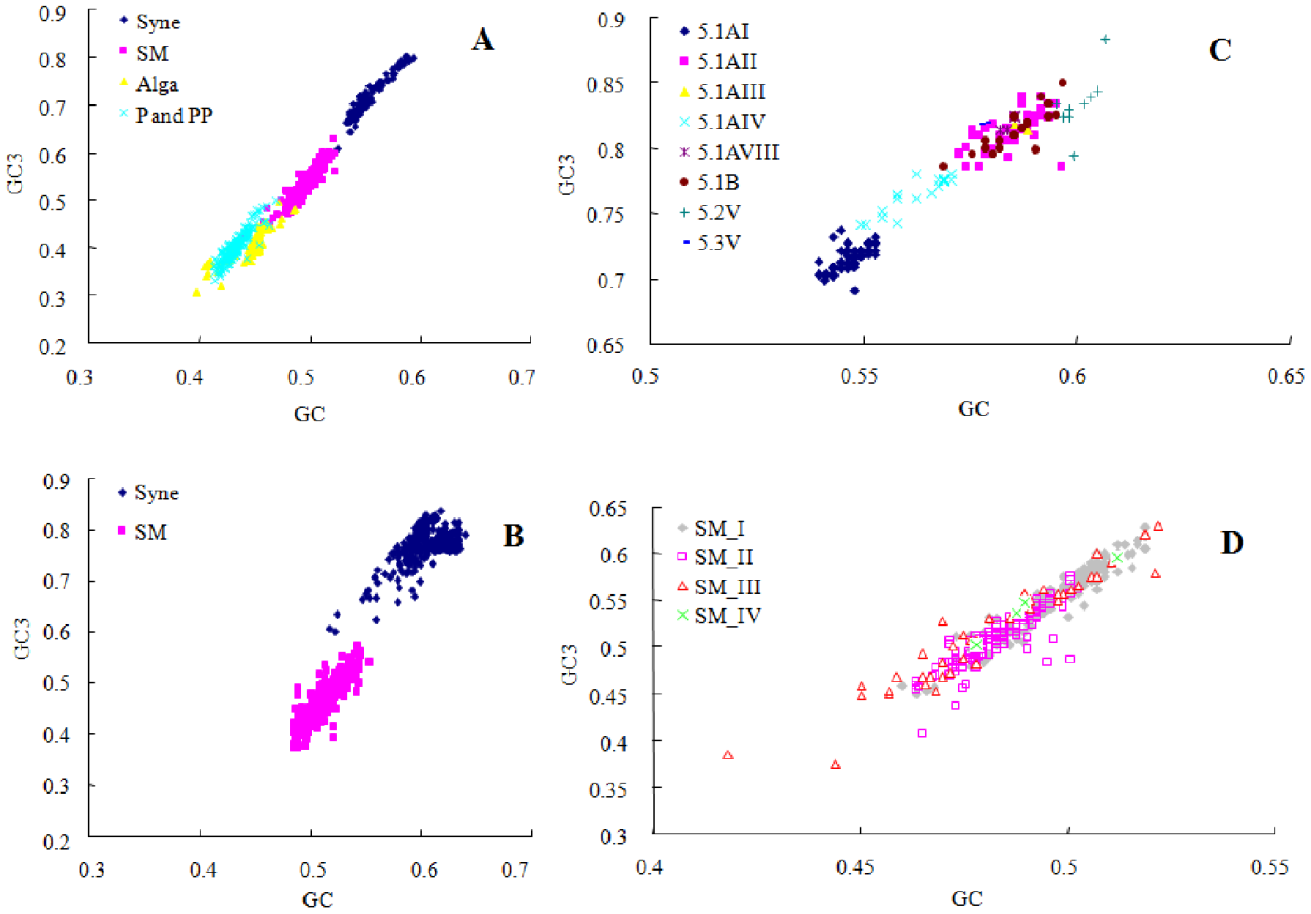
Relationship between the overall GC versus GC3 of *psbA* squences. A. The partitioning characteristics for all communities (data from ECS). *Synechococcus*: y = 2.5655x - 0.7067 (R^2^ = 0.9265); Synechomyovirus: y = 2.463x - 0.6944 (R^2^ = 0.8566). B. The partitioning characteristics for *Synechococcus* and Synechomyovirus (data from GOS database). C. The partitioning characteristics for different *Synechococcus* clades. D. The partitioning characteristics for different Synechomyovirus clades.

The overall average GC content differed substantially among different communities, ranging from 40% to 60%. However, the GC3 content had a much wider range, 30% to 80% (**Fig. 5A**).

As can be seen from **Fig. 5A**, different communities take up their own partitioned area. *Synechococcus* is in the top position, and *psbA* genes in Synechomyoviruses are almost completely separated from their hosts. This clearly separated pattern is also shown in **Fig. 5B**, which sequences are collected from GOS database *Prochlorococcus* and their phages had much lower GC and GC3 content, and were mixed together (**Fig. 5A**). The low-light *Prochlorococcus* was located at the upper end of this region (although some isolated low-light *Prochlorococcus* occupied the lower position of the *Synechococcus* region). The GC content of *psbA* genes in photosynthetic eukaryotic picophytoplankton (PEP) shared a similar range with *Prochlorococcus* and their phages, but their GC3 content was relatively low. In our data, the lowest GC and GC3 contents were also found in PEPs.

The value of GC3 was much higher (8.3%–21.4%) than the corresponding GC value in *Synechococcus psbA* sequences, and the range was from −2% to 10.4% in Synechomyoviruses, while the GC3 value of all *psbA* sequences belonging to *Prochlorococcus* and their myoviruses were usually lower than the GC value except for several low-light *Prochlorococcus* ones.

The main clades of *Synechococcus* also showed obvious partitioning characteristics from the GC and GC3 values (**Fig. 5C**). Subcluster 5.2 (with the highest GC content) occupied the top position, followed by subcluster 5.1B and clade 5.1A II which occupied overlapping regions. Clade 5.1A I had the lowest GC and GC3 content, and occupied the bottom position. Another main clade (5.1A IV) was just between clades 5.1A I and 5.1A II. The clear partitioning showed that GC and GC3 played significant roles in the divergence of *Synechococcus*. Using GC and GC3 partitioning Synechomyoviruses, no clear pattern was found (**Fig. 5D**). This might have been due to the high frequency of *psbA* recombination among the cyanophages and between phages and their hosts, which also leads to patchy GC distribution in *psbA* sequences [27], [28].

The GC3 content contributed 85.5% and 82.1% of the overall GC content variation (or decline) in *Synechococcus* and their myoviruses, respectively. The GC3 content played an important role in shaping the overall GC content and the long-term evolution of the GC content. The variation of GC3 usually represents a synonymous mutation process. Mutation is the engine that drives evolution and adaptation forward in that it generates the variation on which natural selection acts. Decreased genomic GC content, together with decreased bacterial genome size (or gene number) are also found from *Synechococcus* to low-light *Prochlorococcus* then to high-light *Prochlorococcus* [9]. However, it is unclear whether the changes are directly related to certain environmental pressures.

It seemed that *psbA* gene conversion was a biased process that tended towards AT from *Synechococcus* to Synechomyoviruses. There is a large gap between *Synechococcus* and *Prochlorococcus* in the GC and GC3 figure (**Figs. 5A and B**), and the Synechomyoviruses just make it. This implied that Synechomyoviruses might have played important roles in the evolution of the cyanobacteria.

Previous work shows that nutrient availability (mainly nitrogen) may have some potential relationship with the genomic GC content, and the high nitrogen-uptake ability bacteria, for example nitrogen-fixers, tend to hold a higher genomic GC content, since the GC base pairs contained eight N atoms while the AT base pairs had seven N atoms [38]. Viruses assemble their particles on the principle of the least energy and materials, which might have contributed to the low GC content in the Synechomyoviruses. This also provided hints: 1) that *Prochlorococcus* (with its much lower GC content) could live better than *Synechococcus* in the oligotrophic oceanic area; 2) that the high-light *Prochlorococcus*, which usually lives in the surface water with few nutrients, have a lower GC content than the low-light ones [9], [39].

### Codon Usage

A more than double codon usage frequency of *psbA* sequences was found between *Synechococcus* and their myoviruses. The third position nucleotides of the preferred codons for *Synechococcus* were all G and C, while most Synechomyoviral *psbA* sequences (72.7%) had A and T at the third position (**Table 2**). The number of preferred codons for Synechomyoviruses was nearly twice that for *Synechococcus*, which indicated that some rare codons in *Synechococcus* became frequent in the phages. That also showed that different evolutionary or selection pressure occurred between the Synechomyoviruses and their hosts.

**Table 2:** The difference of codon usage.

Codon	Amino acids	Syne/SM*	Codon	Amino acids	Syne/SM*
**AGG**	(Arg/R)	0.00	**CAG**	(Gln/Q)	2.05
GUA	(Val/V)	0.01	**UUG**	(Leu/L)	2.06
CAU	(His/H)	0.02	**CCC**	(Pro/P)	2.75
AGU	(Ser/S)	0.02	**CGC**	(Arg/R)	2.89
CCA	(Pro/P)	0.02	**AGC**	(Ser/S)	3.35
AGA	(Arg/R)	0.05	**GGC**	(Gly/G)	3.75
UUA	(Leu/L)	0.08	**GCC**	(Ala/A)	4.09
ACU	(Thr/T)	0.10	**GUG**	(Val/V)	4.35
UUU	(Phe/F)	0.10			
**GGG**	(Gly/G)	0.12			
CUA	(Leu/L)	0.12			
UAU	(Tyr/Y)	0.15			
AAU	(Asn/N)	0.16			
AUU	(Ile/I)	0.25			
AUA	(Ile/I)	0.25			
CGA	(Arg/R)	0.27			
**GCG**	(Ala/A)	0.31			
**ACG**	(Thr/T)	0.40			
**UCG**	(Ser/S)	0.42			
GCA	(Ala/A)	0.44			
**CCG**	(Pro/P)	0.45			
GAU	(Asp/D)	0.48			

* Syne/SM, the ratio means the codon usage of *Synechococcus psbA* sequences divided by the codon usage in Synechomyovirus. The third codon position with G or C is highlighted using bold/underline.

The diversity and divergence between *Synechococcus* and Synechomyoviruses studied here shed light on the ecology and evolution of phage-host interactions in the environment. The isolation of more phage-host modeling systems is required to better understand their antagonistic co-evolution characteristics. Further environmental metagenomics and transcriptomics with deeper sequencing might give some clues concerning the in-situ dynamics of phage-host ecosystems.

## Materials and Methods

### Sample Collection

Water samples were collected on board during two cruises (from Dec. 2009 to Jan. 2010, and Jun. 2010) (**Fig. 1**). Transect DH3 (including stations DH3-1, DH3-3 and DH3-6) was from the summer cruise. The other two transects, PN (including stations YZE, PN09 and PN04) and DH22-DH24-DH27b, were from the winter cruise. Subsamples (2 to 3 liters) were pre-filtered through a 3-µm filter (GE Water & Process Technologies) and subsequently filtered onto 0.22-µm-pore-size polycarbonate filters (Millipore). The filters were immediately frozen and stored at −80°C until further analysis. All necessary permits were obtained for the described field studies. One permit was required and obtained from the State Oceanic Administration People’s Republic of China.

### Nucleic Acid Extraction

DNA was extracted using the hot sodium dodecylsulphate, phenol: chloroform: isoamyl alcohol, ethanol precipitation extraction protocol as described initially by Fuhrman *et al*. [40] with minor modifications by Zeng *et al*. [41]. The DNA was stored at −20°C for future use.

### Construction of *psbA* Gene Clone Libraries

A polymerase chain reaction (PCR) was performed using the primers, *psbA*-F (5′-GTN GAY ATH GAY GGN ATH MGN GAR CC-3′) and *psbA*-R (5′-GGR AAR TTR TGN GCR TTN CKY TCR TGC-AT-3′) [42]. The PCR reaction mixture (50 µL) consisted of 25 µL ExTaq premix (TaKaRa, Dalian, China), 0.5 µM each primer, and 2 µL (ca. 10 ng DNA) of template. The amplification conditions comprised steps at 95°C for 5 min, 30 cycles at 94°C for 1 min, 55°C for 1 min, and 68°C for 1 min followed by one step of 10 min at 72°C. The amplified products were gel-purified and ligated into the pMD18-T vector (TaKaRa, Dalian, China) and then transformed into competent cells of *Escherichia coli* DH5*a*. The ampicillin-resistant clones were randomly picked and screened for inserts using performing colony PCR with M13 primers (Invitrogen, Shanghai, China) for the vector.

### Sequencing and Phylogenetic Analysis

Clones were sequenced on an ABI 3730 genetic analyser using M13F as the sequencing primer. All *psbA* gene sequences were checked manually based on the BLAST result and given a general classification. All sequences were aligned using the program ClustalX 2 [43]. Phylogenetic trees were constructed using the neighbour-joining algorithms of MEGA software 3.0 [44]. Sequences (both nucleotide and amino acid sequences) were grouped as operational taxonomic units (OTUs) by 99% or greater sequence similarity in the DOTUR program for further rarefaction analyses [45]. Rarefaction curves were calculated based the OTUs results using the statistical program PAST, ver. 1.34 (http://folk.uio.no/ohammer/past).

All partial *psbA* gene sequences obtained in this study have been deposited into the GenBank database under accession numbers: KC997816–KC998700.
